# Trend of hand, foot, and mouth disease from 2010 to 2021 and estimation of the reduction in enterovirus 71 infection after vaccine use in Zhejiang Province, China

**DOI:** 10.1371/journal.pone.0274421

**Published:** 2022-09-20

**Authors:** Haocheng Wu, Ming Xue, Chen Wu, Qinbao Lu, Zheyuan Ding, Xinyi Wang, Tianyin Fu, Ke Yang, Junfen Lin

**Affiliations:** 1 Zhejiang Province Center for Disease Control and Prevention, Hangzhou, Zhejiang Province, China; 2 Key Laboratory for Vaccine, Prevention and Control of Infectious Disease of Zhejiang Province, Hangzhou, Zhejiang Province, China; 3 Hangzhou Centre for Disease Control and Prevention, Hangzhou, Zhejiang, Province, China; The Chinese University of Hong Kong, HONG KONG

## Abstract

**Background:**

Zhejiang, ranked in the top three in HFMD (hand, foot, and mouth disease) incidence, is located in the Yangtze River Delta region of southeast China. Since 2016, the EV71 vaccine has been promoted in Zhejiang Province. This study aimed to investigate the trend and seasonal variation characteristics of HFMD from 2010 to 2021 and estimate the reduction in enterovirus 71 infection after vaccine use.

**Methods:**

The data on HFMD cases in Zhejiang Province from January 2010 to December 2021 were obtained from this network system. Individual information on cases and deaths was imported, and surveillance information, including demographic characteristics and temporal distributions, was computed by the system. The Joinpoint regression model was used to describe continuous changes in the incidence trend. The BSTS (Bayesian structural time-series models) model was used to estimate the monthly number of cases from 2017 to 2021 based on the observed monthly incidence during 2010–2016 by accounting for seasonality and long-term trends. The seasonal variation characteristics of HFMD pathogens were detected by wavelet analysis.

**Results:**

From 2010 to 2021, the annual incidence rate fluctuated between 98.81 cases per 100,000 in 2020 and 435.63 cases per 100,000 in 2018, and 1711 severe HFMD cases and 106 fatal cases were reported in Zhejiang Province, China. The annual percent change (APC) in EV71 cases was -30.72% (95% CI: -45.10 to -12.50) from 2016 to 2021. The wavelet transform of total incidence and number of cases of the three pathogens all showed significant periodicity on the 1-year scale. The average 2-year scale periodicity was significant for the total incidence, EV71 cases and Cox A16 cases, but the other enterovirus cases showed significant periodicity on the 30-month scale. The 6-month scale periodicity was significant for the total incidence, EV71 case and Cox A16 case but not for the other enteroviruses case. The relative error percentage of the performance of the BSTS model was 0.3%. The estimated number of cases from 2017 to 2021 after the EV-A71 vaccines were used was 9422, and the reduction in the number of cases infected with the EV71 virus was 73.43% compared to 70.80% when the impact of the COVID-19 epidemic in 2020 was excluded.

**Conclusions:**

Since 2010, the incidence of EV71 infections has shown an obvious downward trend. All types of viruses showed significant periodicity on the 1-year scale. The periodicity of the biennial peak is mainly related to EV71 and Cox A16 before 2017 and other enteroviruses since 2018. The half-year peak cycle of HFMD was mainly caused by EV71 and Cox A6 infection. The expected incidence will be 2.76 times(include the cases of 2020) and 2.43 times(exclude the cases of 2020) higher than the actual value assuming that the measures of vaccination are not taken. EV71 vaccines are very effective and should be administered in the age window between 5 months and 5 years.

## Introduction

Hand, foot and mouth disease (HFMD) is a common global infectious disease caused by various enteroviruses, primarily in infants and children aged younger than 5 years, and outbreaks or epidemics often occur [1–5]. Clinically, HFMD is mainly characterized by fever and rash or herpes on the palms, soles, and oral mucosa [6, 7]. The course of the disease is self-limiting, the symptoms are generally mild, and the prognosis is good. However, a few patients can be complicated by aseptic meningitis, encephalitis, acute flaccid paralysis, respiratory infection and myocarditis, and some patients may even die [8, 9]. Among these enteroviruses, enterovirus 71 (EV71) is a major pathogenic agents that often causes severe complications [10]. EV71 began circulating in the Netherlands in 1963 and was first isolated in California, USA, in 1969 [11]. Since then, EV71 has been detected in a range of countries worldwide, and outbreaks in recent years have mainly occurred in the Asia-Pacific region, including China, Malaysia, and Japan [10, 12, 13]. In China, hand, foot and mouth disease has caused a major disease burden. Since the outbreak of EV71 in Anhui in May 2008, HFMD was included in Class C notifiable diseases, and its morbidity and mortality have been at the forefront of notifiable infectious diseases [14]. Zhejiang, ranked in the top three in the HFMD incidence, is located in the Yangtze River Delta region of southeast China. The number of reported cases increased rapidly in 2009–2016, ranking first among notifiable diseases, and EV71 was the predominant serotype [15]. Since 2016, three inactive monovalent EV-A71 vaccines have been approved for market entry in China [16, 17]. This vaccine also began to be promoted at the same time in Zhejiang Province. Some studies have shown a highly effective protection rate for HFMD cases and the vaccine has performed well in severe HFMD cases in a real-world setting [18–20]. This study aimed to investigate the trend of hand, foot, and mouth disease from 2010 to 2021. The Bayesian structural time series (BSTS) model was applied to estimate the reduction in enterovirus 71 infection after vaccine use. We also used joinpoint regression analysis to observe the trend in the EV71 incidence. The seasonal variation characteristics of HFMD pathogens were detected by wavelet analysis.

## Materials and methods

### Ethical review

This study was reviewed and approved by the Ethics Committee of the Zhejiang Provincial Centers for Disease Control and Prevention. All the data of the individuals were kept confidential as requested. Verbal informed consent was obtained from adult cases and from the parent/guardian for the pediatric patients before diagnosis and reporting to the China Information Network System of Disease Prevention and Control. All the methods employed in the study were in accordance with the applicable guidelines and regulations.

### Profile of Zhejiang Province

Zhejiang Province is located in southeast China between longitudes 118°E-123°E and latitudes 27°N-32°N. There are two subprovincial cities (Hangzhou and Ningbo) and nine prefecture-level cities, including Wenzhou, Huzhou, Jiaxing, Shaoxing, Jinhua, Zhoushan, Quzhou, Taizhou and Lishui, which cover 90 counties.

### Data collection

Since 2008, any HFMD case diagnosed in the hospital must be reported through the China Information Network System of Disease Prevention and Control by the medical staff. The data of the HFMD cases in Zhejiang Province from January 2010 to December 2021 and the population data were obtained from this network system. Virological surveillance was conducted by the local CDC. Specimens were collected from some cases and sent to laboratories for coxsackie virus A 16 (CV-A16) and enterovirus 71 (EV71) and other enterovirus nucleic acid testing by polymerase chain reaction or virus isolation [16]. Individual information on cases and deaths was imported, and surveillance information, including demographic characteristics and temporal distributions, was computed by the system. The definition of reporting cases referred to the ‘Guidelines for Prevention and Control of Hand, Foot and Mouth Disease’ (Version 2009) of China.

### Joinpoint regression

Joinpoint regression is a model used to describe continuous changes in the incidence trend. The grid-search method was used to fit the regression function, with unknown joinpoints assuming constant variance and uncorrelated errors [21]. The joinpoint regression model for the observations (*x*_1_, *y*_1_),…, (*x*_*n*_, *y*_*n*_), (*x*_1_≤…≤*x*_*n*_) without a loss of generality is written as

$$E[y/x]=\beta_{0}+\beta_{1}+\delta_{1}{\left(x-\tau_{1}\right)}^{+}+\ldots +\delta_{k}{\left(x-\tau_{k}\right)}^{+},$$ (1)

where y is the outcome of interest, x is the time variable, τk's are the unknown joinpoints, and *α*^+^ = *α* for *α* > 0 and 0 otherwise.

The approximate permutation test was used to find the number of significant joinpoints, each p value was obtained using Monte Carlo methods, and the overall asymptotic significance level was maintained using Bonferroni correction. The objective indicator was the annual percent change (APC) of each period segment, estimated according to the following formula:

$$APCi=[(Exp(\beta_{i})-1]\times 100,$$ (2)

where *β*_*i*_ represents the slope of the period segment [21, 22].

### Bayesian structural time-series models (BSTS)

The BSTS model comprises three main components: the Kalman filter, spike-and-slab method, and Bayesian model averaging [23, 24]. This model is a state-space model that makes it possible to infer the temporal evolution of attributable impact, to incorporate empirical priors on the parameters in a fully Bayesian treatment and to accommodate multiple sources of variation, including local trends, seasonality and the time-varying influence of contemporaneous covariates [23]. We used the BSTS model to estimate the monthly number of cases from 2017 to 2021 based on the observed monthly incidence during 2010–2016 by accounting for seasonality and long-term trends. The BSTS model is written as:

$$Log(y_{t})=Z_{t}^{T}\alpha_{t}+\mu_{t}+\tau_{t}+\varepsilon_{t}\;\varepsilon_{t}∼N(o,H_{t})$$ (3)

$$\alpha_{t+I}=T_{t}\alpha_{t}+R_{t}\eta_{t}\ldots \eta_{t}∼N(o,Q_{t})$$ (4)

Eq (3) is the observation equation, which links the observed data *y*_*t*_ to a latent d-dimensional state vector *α*_*t*_. $Z_{t}^{T}$is a *d*-dimensional output vector, *μ*_*t*_ is used to control the long-term trend, and *τ*_*t*_ is used to control the seasonality with a variable of month (12 per year). Eq (4) is the state equation, which governs the evolution of the state vector *α*_*t*_ over time [23]. *T*_*t*_ is a d × d transition matrix, and *R*_*t*_ is a d × q control matrix.

*ε*_*t*_ and *η*_*t*_ are the randomly and independently distributed Gaussian error terms with zero mean and variance *H*_*t*_ and *Q*_*t*_, respectively. The model is implemented with the Markov chain Monte Carlo (MCMC) algorithm using 1000 iterations [23, 24]. The formula of relative reduction is written as:

$$\text{Relative}\;\text{reduction}\;\left(\text{\%}\right)=100\%\times \left(\text{number}\;\text{of}\;\text{expected}\;\text{cases}\;-\text{number}\;\text{of}\;\text{observed}\;\text{cases}\right)/\text{number}\;\text{of}\;\text{expected}\;\text{cases}.$$ (5)

### Wavelet analysis [25]

Wavelet analysis can effectively reveal the time-frequency structure characteristics of a nonstationary time series. The common method of wavelet analysis is wavelet transform. The wavelet transform is a theory developed in the 1980s. Its function is similar to classical Fourier analysis, but the wavelet base is used instead of the sine wave base. The wavelet transform includes two features: "adaptability", which can automatically adjust relevant parameters according to the object analyzed by wavelet, and "mathematical microscope property", which can automatically "focus" according to the observed object. The basic method of wavelet transform is to select the function that satisfies the time domain integral to zero as the basic wavelet and generate a family of functions by stretching and translating the basic wavelet. The projection on the frame is decomposed.

The wavelet function ψ(*t*) is called the basic wavelet, and the function family {ψ_*a*,*b*_} is generated by the translation and expansion of ψ(*t*).


$$\Psi_{a,b}=\frac{1}{\sqrt{a}}\Psi (\frac{t-b}{a})\;a,b\in R,a\neq 0$$ (6)

In Eq (6), *a* is the scale factor, which is a coefficient concerning the scale, and *b* is a translation factor, which is a factor concerning the event. Next, the continuous wavelet transform of the signal *f*(*t*) is defined as:

$$Wf(a,b)\leq f,\Psi_{a,b}\geq f(t)\int_{-\infty }^{+\infty }\Psi_{a,b}(t)f(t)dt$$ (7)

### Statistical analysis

The joinpoint regression model was used to examine the trend of the incidence of HFMD from 2010 to 2021 by Joinpoint software (version 4.5.0.1). The BSTS model and wavelet analysis were run by R Studio (version 1.2.5001). A *P* value less than 0.05 represented statistical significance for all the tests.

## Results

### Incidence trend

The incidence rate of hand, foot and mouth disease is characterized by a peak every other year. From 2010 to 2021, the annual incidence rate fluctuated between 98.81 cases per 100,000 in 2020 and 435.63 cases per 100,000 in 2018. From January 2010 to December 2021, 1711 severe HFMD cases and 106 fatal cases were reported in Zhejiang Province, China. Additionally, 1004, 244, 112, 44, 152, 25, 88, 18, 14, 1, 4 and 5 severe cases were identified in each year of the last twelve years. Furthermore, 37, 24, 17, 3, 14, 1, 7, 2, 0, 0, 0 and 1 fatalities occurred each year. A fluctuating reduction in the proportion of cases younger than 5 years was observed from 2010 to 2021, with the highest of 95.46% in 2013 and the lowest of 89.10% in 2021.

**Fig 1:**
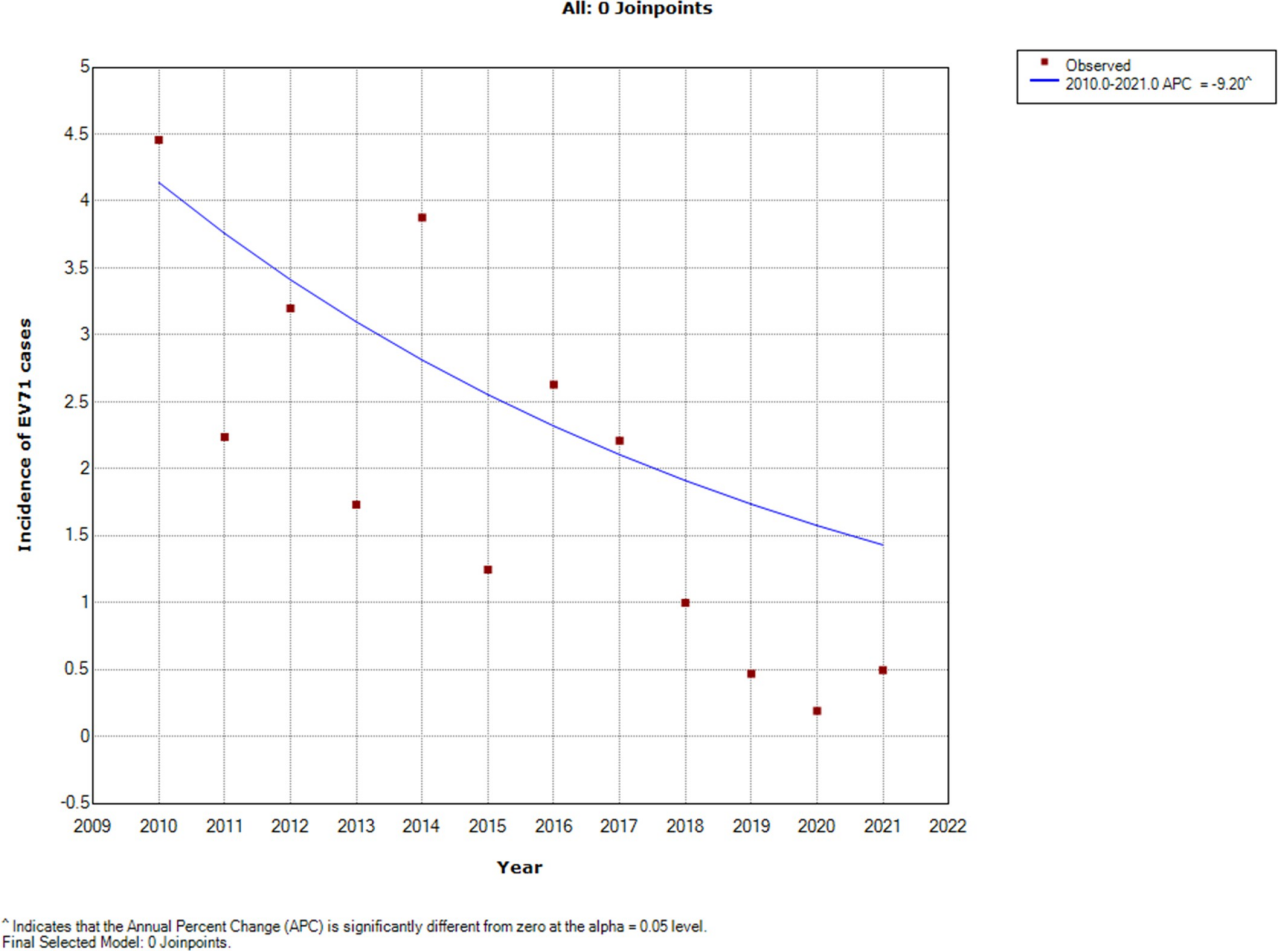
Trend of the incidence of EV71 cases between 2016 and 2021 shown by joinpoint regression. The red squares denote the observed values of the incidence, and the blue line is the slope of the APC. The APC was the annual percent change.

The average numbers of EV71, Cox A16 and other enterovirus cases from 2010 to 2021 were 1083, 954 and 2570, respectively (Table 1). The annual percent change (APC) of the incidence of EV71 cases was -9.20% (95% CI: -16.70 to -1.10) from 2016 to 2021, a value that was significantly different from zero at the alpha = 0.05 level (test statistic = -2.50, *P* = 0.025), indicating a monotone decreasing trend in the incidence (Fig 1). The APC values of the incidence of total case, Cox A16 case and other enterovirus cases were -2.20% (95%CI:6.30 to 11.40), -0.40% (95%CI:-7.00 to 6.70) and 5.80%%(95%CI:5.70 to 18.70), respectively, which are all not significantly different from zero at the alpha = 0.05 level (test statistic = 0.60, *P* = 0.60, test statistic = -0.10, *P* = 0.90, test statistic = 1.10, *P* = 0.30,respectively).

**Table 1:** Virological surveillance of HFMD from 2010 to 2021.

Year	Incidence of EV71 cases(1/100,000)	Incidence of Cox A16 cases(1/100,000)	Incidence of Other enteroviruses cases(1/100,000)
**2010**	4.46	2.50	1.16
**2011**	2.24	0.73	0.84
**2012**	3.20	2.51	2.34
**2013**	1.73	0.71	5.38
**2014**	3.88	2.63	5.23
**2015**	1.25	1.47	4.95
**2016**	2.63	2.80	5.15
**2017**	2.21	0.95	4.71
**2018**	1.00	2.14	10.58
**2019**	0.47	2.84	4.92
**2020**	0.19	0.21	4.46
**2021**	0.50	1.09	4.73

### Seasonal and periodiccity characteristics

**Fig 2:**
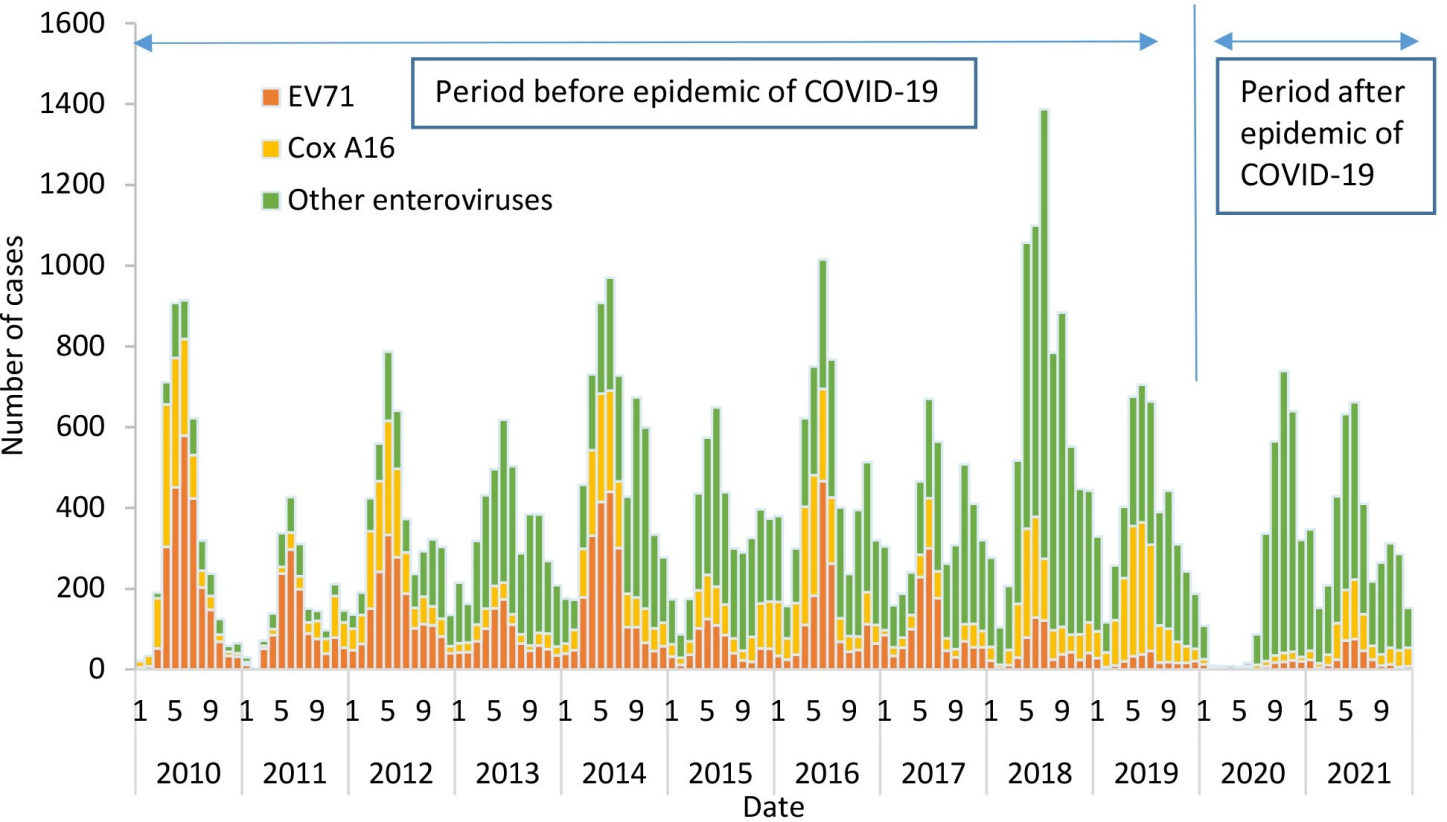
Seasonal distribution trends of EV71, Cox A16 and other enterovirus cases.

This disease showed semiannual peaks of activity, including a major peak from May to June followed by a smaller peak in autumn. From May to June, the average proportions of EV71 cases, Cox A16 cases and other enteroviruses cases were 40.01%, 35.73% and 21.69%, respectively. From September to November, the average proportions of the three pathogens were 13.99%, 14.79% and 32.41%, respectively (Fig 2). In 2020, after the outbreak of the novel coronavirus disease (COVID-19), this seasonal characteristic was changed, and gradually returned to the previous seasonal characteristic in 2021.

**Fig 3:**
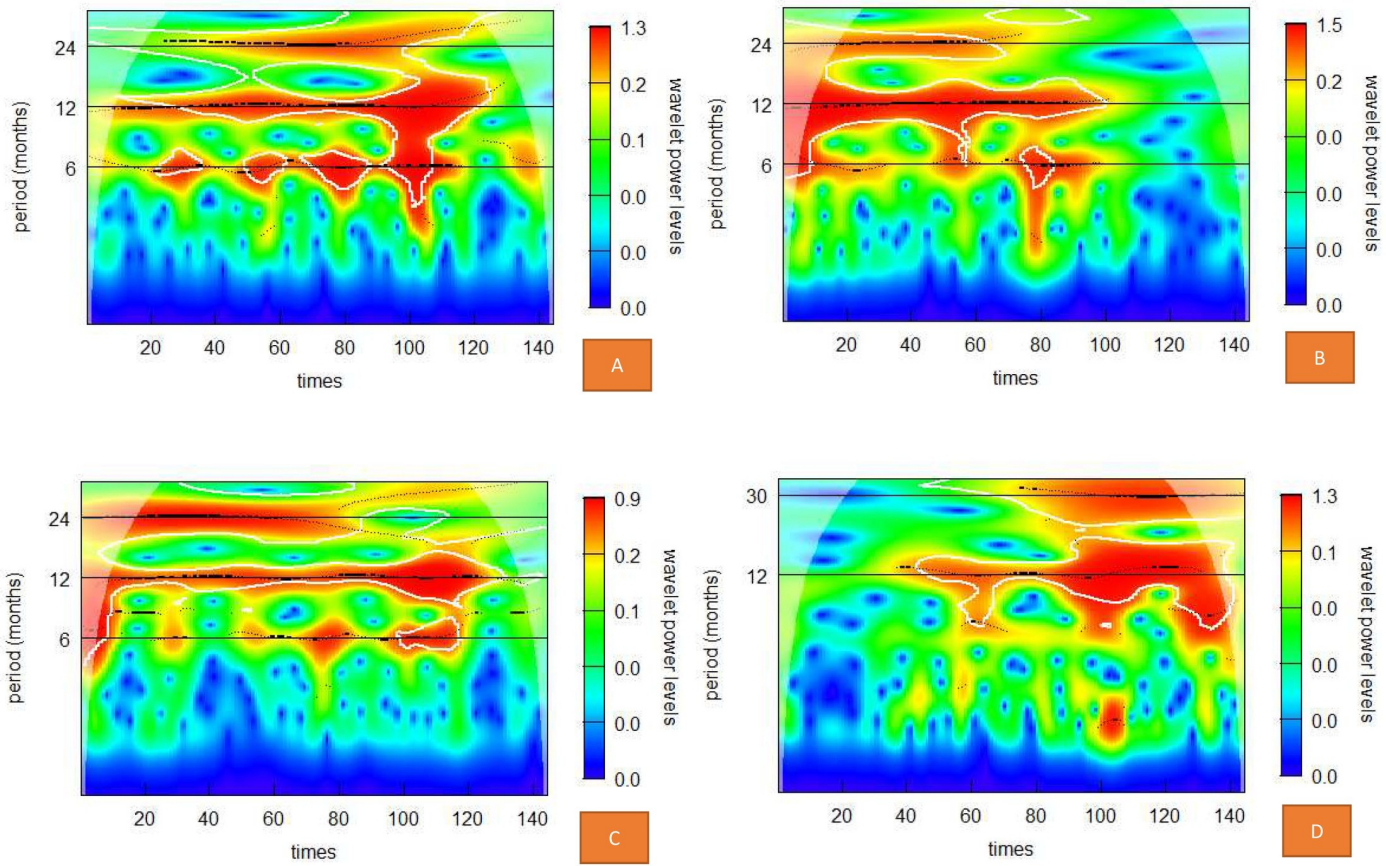
Wavelet spectrum of HFMD for total cases, EV71 cases, Cox A16 cases and other enterovirus cases. (A) Wavelet spectrum of the total case. (B) Wavelet spectrum of the EV71 case. (C) Wavelet spectrum of Cox the A16 case. (D) Wavelet spectrum of other enteroviruses.

**Fig 4:**
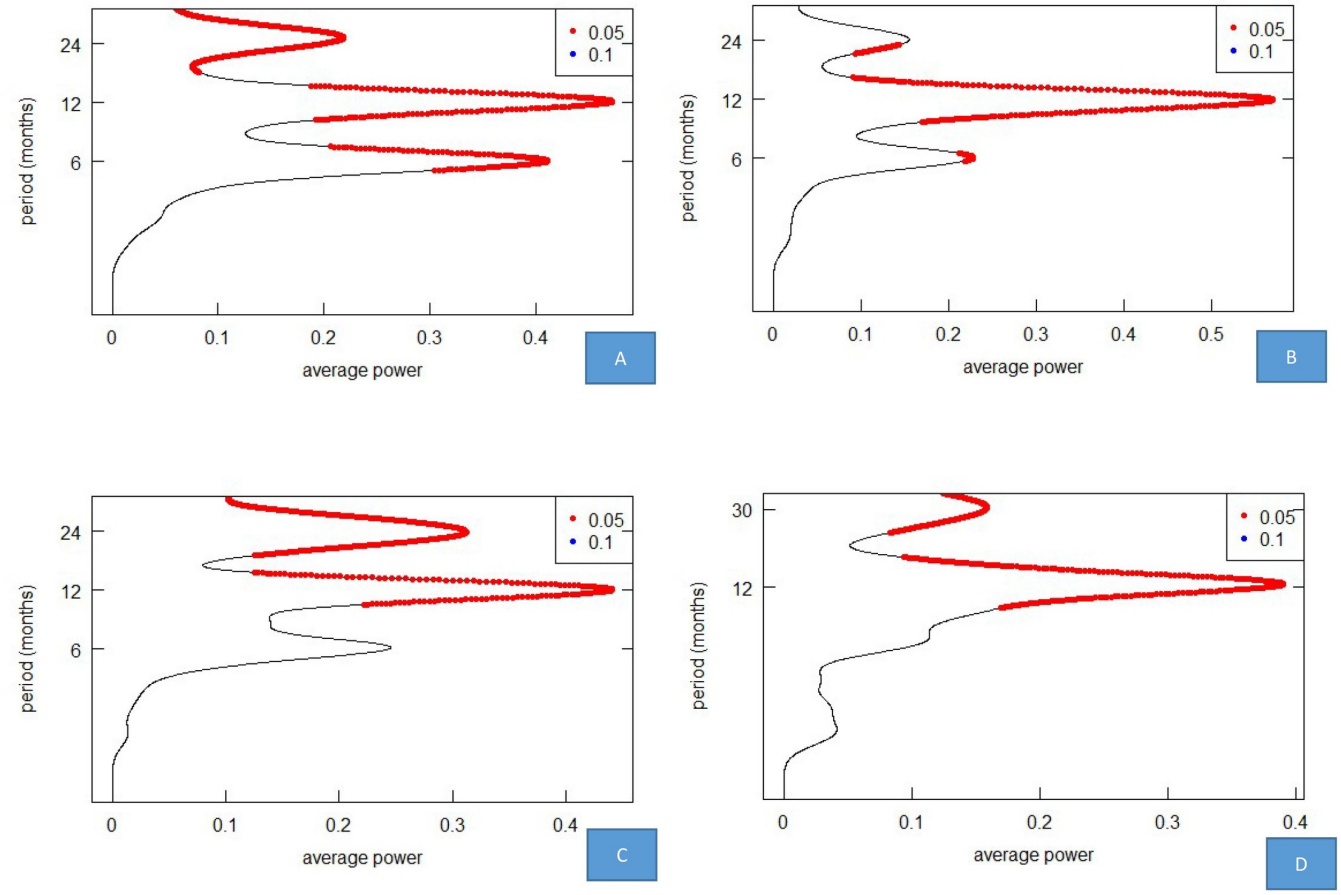
The average power of HFMD for the total case, EV71 case, Cox A16 case and Other enteroviruses cases. (A) Wavelet spectrum of the total case. (B) Wavelet spectrum of the EV71 case. (C) Wavelet spectrum of Cox A16 case. (D) Wavelet spectrum of other enteroviruses.

Figs 3 and 4 show the wavelet power spectrum for the total case, EV71 case, Cox A16 case and Other enteroviruses case. The wavelet transform of total incidence and the number of cases of the three pathogens all showed significant periodicity on the 1-year scale. The average power was also significant on the 1-year scale. The average 2-year scale periodicity was significant for total incidence, EV71 cases and Cox A16 cases, but the other enterovirus cases showed significant periodicity on the 30-month scale. The 6-month scale periodicity was significant for total incidence, EV71 case and Cox A16 case, but not significance for the Other enteroviruses case. The average power was only significant for EV71 cases on the 6-month scale. From 2018, the periodicity of EV71 cases was not significant at the 1-year scale, 2-year scale and 6-month scale. There was no significance for Cox A16 cases on a 2-year scale from 2017. After 2018, the 24- to 30-month scale was mainly attributed to the other enterovirus cases.

### Estimation of the reduction in enterovirus 71 infection

**Fig 5:**
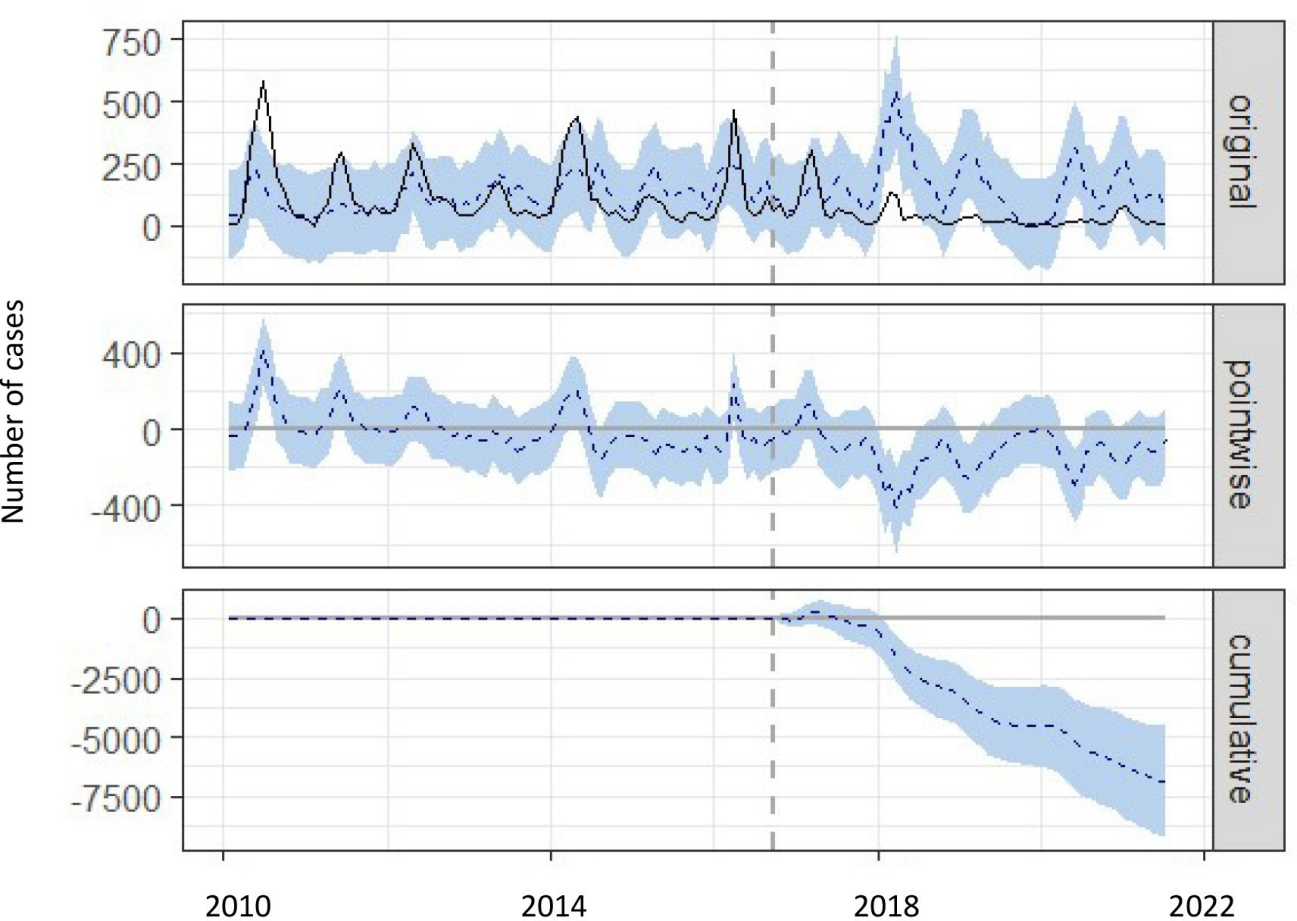
The monthly counts of HFMD during 2010–2021 and the comparison with expected infection among 2017–2021 in Zhejiang Province, China. The first panel shows the data and a counterfactual prediction for the posttreatment period. The second panel shows the difference between observed data and counterfactual predictions. The third panel sums the pointwise contributions from the second panel, resulting in a plot of the cumulative effect of the intervention.

Before 2017, a total of 10498 cases of hand, foot and mouth disease infected with EV71 virus were reported in Zhejiang Province. The fitted mean number of EV71 in this period was 10467 (95% confidence interval: 762–25338), and the relative error percentage of the modeling performance was 0.3%. There were 2503 cases of hand, foot and mouth disease infected with EV71 virus reported from 2017 to 2021 in Zhejiang Province (Fig 5). The estimation of the mean number of cases in this period after the EV-A71 vaccines were used was 9422 (95% confidence interval: 1833–20420), and the reduction in the number of cases infected with EV71 was 73.43%. There were 2390 cases of hand, foot and mouth disease infected EV71 virus reported among 2017 to 2021(exclude the cases of 2020) in Zhejiang Province. The estimation of the mean numbers of case in this period(exclude the cases of 2020) after the EV-A71 vaccines used was 8186(95% confidence interval:1554–17030), the reduction in the number of cases infected with EV71 virus were 70.80%.

## Discussion

After 2010, the incidence of EV71 infections showed an obvious downward trend, but a major reduction was not identified in the total incidence, Cox A16 and other enteroviruses cases. Especially after 2018, the incidence of EV71 is less than 1 cases per 100,000. This result is similar to other studies; one of the important reasons was the lack of a cross-protection effect of vaccines against various virus strains [16, 26, 27]. The fluctuation of the incidence of the total cases was due to steady birth rate, and non-EV71 enteroviruses contributed to the increase in incidence rate [16]. Similar to the decrease in EV71 cases, the case severity and fatality rates have also been obviously reduced. The decrease in case severity and fatality was primarily attributed to reduction of the EV71 virus, and similar findings have been reported in some other cities in China [28, 29]. However, the reduction in severe and fatal HFMD cases may also be related to the improvement of medical technology. Many cases were diagnosed at an early stage of severe disease, and prompt interventions were implemented, thus reducing the incidence of severe and fatal cases. It was reported that young children were at a high risk of infection with HFMD, particularly children 1–5 years of age [30]. In our study, the proportion of individuals aged 0–5 years decreased in 2017–2021, and the age distribution shifted to older age, which was similar to a previous study [17]. One reason possibly driving this age shift was the waning of vaccine-derived immunity, and the other reason may be the decline in the risk of infection in the high-risk population after implementation of EV-A71 vaccination [17, 31].

Similar to previous studies, the incidence of HFMD showed obvious seasonal and cyclical characteristics [32]. Our research shows that there are three main types of HFMD incidence cycles, which are the peak incidence cycles of half a year, one year and every other year. Overall, the drivers of hand, foot, and mouth disease periodicity are not fully understood, but this might be complicated by interference between the causative enterovirus serotypes and associated with climatic factors, including precipitation, sunshine, temperature, and air pressure [32]. Our study suggests that the periodic characteristics of HFMD may be related to the peak incidence of each pathogen. The viruses EV71, Cox A16 and other enteroviruses all showed significant periodicity on the 1-year scale and exhibited summer peaks. The periodicity of the biennial peak is mainly related to EV71 and Cox A16 before 2017. However, beginning in 2018, the other enteroviruses gradually replaced the other two viruses as the main contributors to the biennial peak, and this cross-year cycle was extended to 30 months. Because most non-EV71/non-Cox A16 enteroviruses were not identified for specific serotypes, the current predominant viral strain is not well understood [16]. The whole etiological spectrum following EV71 and Cox A6 was CV -A6, CV -A16, CV- A10, CV-A5, CV -A2 and so on. The year 2020 is a special period. Due to the impact of nondrug intervention measures (including restricting crowd gatherings, delaying school openings, etc.) In the COVID-19 epidemic, the incidence of other infectious diseases dropped significantly and gradually recovered in 2021 [24]. For HFMD, the incidence peak of 2021 shifted to October to November, which may be one reason for the 30-month cross-year cycle. Our research also shows that the half-year peak cycle of hand, foot and mouth disease was mainly caused by EV71 and Cox A6 infection, and other types of viruses do not seem to exhibit this cycle. Especially after 2017, with the sharp decline in the incidence of EV71, the characteristics of the small peak incidence in the half-year cycle were obviously weakened. However, this may also be related to the COVID-19 epidemic and requires more observations in the future for further verification.

Our current and many previous studies suggest that EV-A71 vaccination plays a vital role in reducing EV-A71 infection and decreasing fatal and severe HFMD cases caused by EV-A71 [17, 26, 33]. According to the estimation of this study, if the measures of vaccination are not taken and the incidence of EV-A71 infection develops similarly to the previous epidemic characteristic, the expected incidence will be 2.76 times higher than the actual value. Furthermore, the estimated reduction in the number of cases infected with EV71 virus was 70.80% when the impact of the COVID-19 epidemic in 2020 was excluded. One meta-analysis about the seroprevalence of EV71 antibody in Chinese children suggested that younger infants are likely to be protected by maternal antibody against EV71 infection and become susceptible to the infection since 5 months of age because of the immunity waned and that EV71 infection is more likely to occur in young children at 2–4 years of age [31]. Our study suggested that EV71 vaccines were very effective and should be administered in the window between 5 months and 5 years old.

In conclusion, since 2016, the incidence of EV71 infections has shown an obvious downward trend, but this trend was not identified in the total incidence, Cox A16 and other enterovirus cases. Furthermore, the age distribution shifted to older ages. The incidence of HFMD showed obvious seasonal and cyclical characteristics. All types of viruses showed significant periodicity on the 1-year scale. The periodicity of the biennial peak is mainly related to EV71 and Cox A16 before 2017 and other enteroviruses since 2018. The half-year peak cycle of HFMD was mainly caused by EV71 and Cox A6 infection. According to the BSTS model, the expected incidence will be 2.76 times(include the cases of 2020) and 2.43(exclude the cases of 2020) higher than the actual value assuming that the measures of vaccination are not taken and the epidemic characteristic of EV-A71 infection is similar to the previous times. EV71 vaccines were very effective and should be administered in the window between 5 months and 5 years old.

### Limitation

Several limitations should be noted within our study. First, different from case surveillance, etiological typing cannot be carried out in all cases, so the typing data only came from the surveillance of a portion of cases, which may lead to bias to some degree. Second, our data included cases of HFMD reported from a passive surveillance system. Future studies should consider mild and asymptomatic cases that do not seek medical care. Third, data regarding the influencing variables, including vaccination rate socioeconomic status and climate factors, were not collected. The association between incidence and influencing factors was not analyzed, and this needs to be carried out in future research.

## Supporting information

S1 File Database of the monthly distribution of HFMD cases in Zhejiang Province. (XLSX)
